# Open arterial reconstruction of multiple hepatic artery aneurysms in a patient with hereditary hemorrhagic telangiectasia: A case report

**DOI:** 10.1097/MD.0000000000005430

**Published:** 2016-11-18

**Authors:** Hirotsugu Ozawa, Takao Ohki, Yuji Kanaoka, Koji Maeda, Shin Hagiwara

**Affiliations:** Division of Vascular Surgery, Department of Surgery, Jikei University School of Medicine, Tokyo, Japan.

**Keywords:** case report, hepatic artery aneurysm, hereditary hemorrhagic telangiectasia, open arterial reconstruction, vascular malformation

## Abstract

**Background:**

Hereditary hemorrhagic telangiectasia (HHT) is characterized by mucocutaneous telangiectasia and visceral vascular malformations (VMs). Liver involvement with VMs may lead to high-output cardiac failure, portal hypertension, and biliary disease. There is no curative treatment for the disease, and liver transplantation is indicated for life-threatening complications. Herein, we report a case of multiple hepatic artery aneurysms (HAAs) in a patient with HHT in which open arterial reconstruction was performed. There have only been a few case reports on HAA occurring with HHT. Thus, this case provides important information for the management of HHT-associated HAAs.

**Case summary:**

A 62-year-old female with known HHT was referred to our facility to seek further treatment for a giant HAA. She denied any symptoms except recurrent epistaxis. A computed tomography (CT) scan revealed a right HAA with a diameter of 72 mm, in addition to 2 other minor HAAs. The CT scan also revealed the VMs that were scattered in the liver, and a continuously dilated and tortuous artery existing from the celiac trunk to the right and left hepatic arteries. We performed open arterial reconstruction of the HAAs. Her postoperative course was uneventful.

**Conclusions:**

When treating HAAs, there are a variety of options. However, hepatic VMs might affect HHT patients in various ways postprocedurally. Ligation and embolization of the hepatic artery may lead to complications, such as massive hepatic necrosis. Hepatectomy should be avoided if possible, because a postoperative hyperperfusive state in the remaining liver can cause adverse events. We believe that arterial reconstruction of HHT-associated HAAs might reduce the risk of postprocedural complications with minimal hemodynamic changes in the liver, thus obviating the need for hepatectomy or liver transplantation.

## 1 Introduction

Hereditary hemorrhagic telangiectasia (HHT) is a rare autosomal dominant disease, characterized by mucocutaneous telangiectasia and vascular malformations (VMs) in the major organs such as the lung, gastrointestinal tract, liver, and/or brain.^[1]^ Liver involvement in HHT is typically characterized by VMs (arteriovenous, arterioportal, and/or portovenous malformations). Hepatic VMs, which are usually asymptomatic, can potentially lead to high-output cardiac failure, portal hypertension, and biliary disease.^[2]^ There is no curative treatment for the disease, and liver transplantation is indicated for these life-threatening complications.

Although the presence of dilated and tortuous hepatic vasculature has been frequently described as other imaging findings in patients with hepatic HHT,^[2–4]^ there have been a few case reports on hepatic artery aneurysm (HAA) occurring with hepatic HHT.^[3–6]^ In addition, to the best of our knowledge, there were no cases of arterial reconstruction mentioned in these reports. Herein, we report a case of multiple HAAs in a patient with hepatic HHT in which open arterial reconstruction was effective and obviated the need for major hepatectomy or liver transplantation.

## 2 Case report

Written informed consent was obtained, and the Institutional Review Board approved this case report.

**Figure 1:**
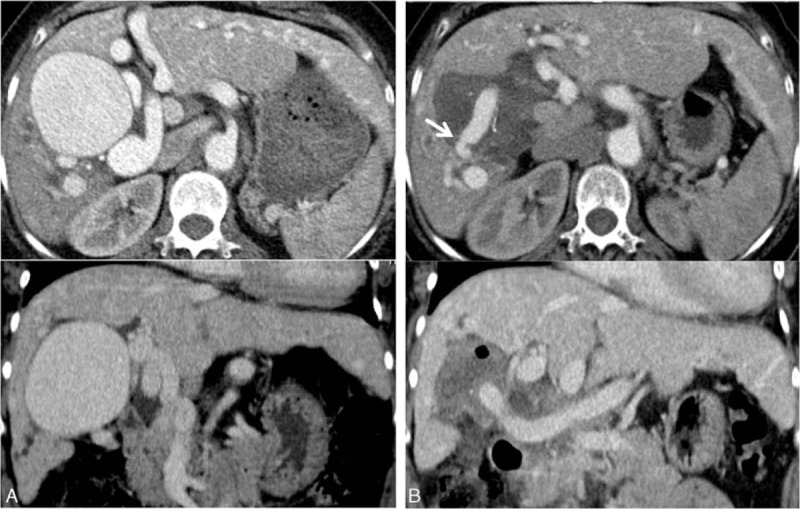
(A) Preoperative CT scan shows a giant right HAA measuring 72 mm in diameter, with a dilated tortuous hepatic vasculature, in addition to VMs scattered predominantly in the left lobe. (B) Postoperative CT scan shows disappearance of the aneurysms and adequate hepatic blood flow with a patent distal anastomosis (arrow) as well as the absence of hepatic atrophy and necrosis. CT = computed tomography, HAA = hepatic artery aneurysm, VMs = vascular malformations.

A 62-year-old female with known HHT was referred to our facility to seek further treatment including possible liver transplantation for a giant HAA. She denied any symptoms except recurrent epistaxis. The patient had been diagnosed with HHT at the age of 56, when presenting with recurrent epistaxis and hematochezia. Definite diagnosis of HHT had been made based on 3 of the 4 Curaçao criteria (the presence of hepatic VMs revealed by abdominal ultrasound imaging, in addition to recurrent epistaxis and skin telangiectasia on her fingers). Because her hepatic VMs were asymptomatic, she had had no previous medical, radiological, or surgical management of her disease. On physical examination, multiple telangiectases were noted on her fingers. Laboratory examination was significant for elevated levels of alkaline phosphatase, 968 g/dL and gamma-glutamyl transpeptidase, 481 U/L. She was classified as Child-Pugh class A (total bilirubin, 0.8 mg/dL, and international normalized ratio, 1.0). Computed tomography (CT) scan revealed 2 right HAAs (72 × 63 mm and 29 × 28 mm) and a common HAA (28 × 27 mm). The larger right HAA was located in the liver bed and just proximal to the bifurcation of the anterior and posterior segmental branches of the right hepatic artery. The smaller right HAA gave off the A6 (the artery feeding S6), and the common HAA gave off the gastroduodenal artery (GDA). In addition, continuously dilated and highly tortuous arteries running from the celiac trunk to the right and left hepatic arteries were noted (Fig. 1A). The biliary duct was dilated in the right lobe, seemingly due to compression of the larger right HAA. Patchy lesions with early enhancement were scattered predominantly in the left lobe, and the middle and left hepatic veins were also enhanced in the early phase. These findings suggested the presence of arteriovenous shunts in the left lobe. Additionally, the left lobe was significantly enlarged, measuring 1105 mL in volume on the basis of CT liver volumetry, whereas the right lobe measured 726 mL.

In summary, it was clear that this was a case of HAAs occurring with asymptomatic hepatic HHT, in which the aneurysms were thought to be a critical clinical feature and surgery was indicated to prevent aneurysmal rupture.

Ahead of a definitive procedure, the patient underwent a diagnostic celiac angiogram via a transfemoral approach. Thereby, 1 outflow was identified from the larger right HAA that seemed to be suitable for reconstruction. Liver transplantation was not considered because her hepatic VMs were asymptomatic and the patient's clinical condition was not sufficiently severe to justify such a procedure. Hepatectomy was also ruled out in order to preserve liver parenchyma. Thus, we decided to perform open arterial reconstruction.

**Figure 2:**
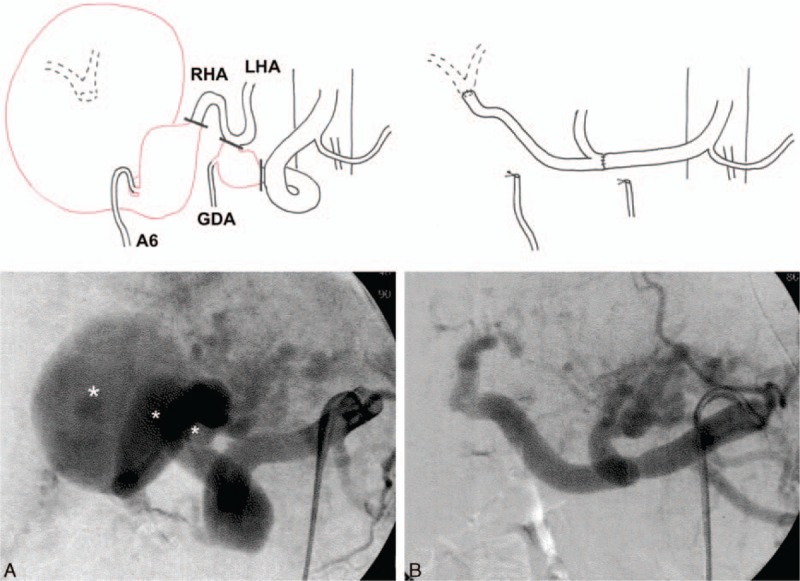
Pre- and postoperative schemas of the hepatic artery anatomy are shown above the angiography. The 3 HAAs are labeled with asterisks. Vessels that were resected are depicted with red lines. Bold lines in the preoperative schema indicate the sites where the hepatic artery was transected. (A) Preoperative angiography shows 2 right HAAs and a common HAA with a dilated tortuous hepatic vasculature, in addition to VMs located in the left lobe. (B) Completion angiography reveals the reconstructed hepatic arteries via direct suturing. A6 = the artery feeding S6, GDA = gastroduodenal artery, HAA = hepatic artery aneurysm, LHA = left hepatic artery, RHA = right hepatic artery, VMs = vascular malformations.

**Figure 3:**
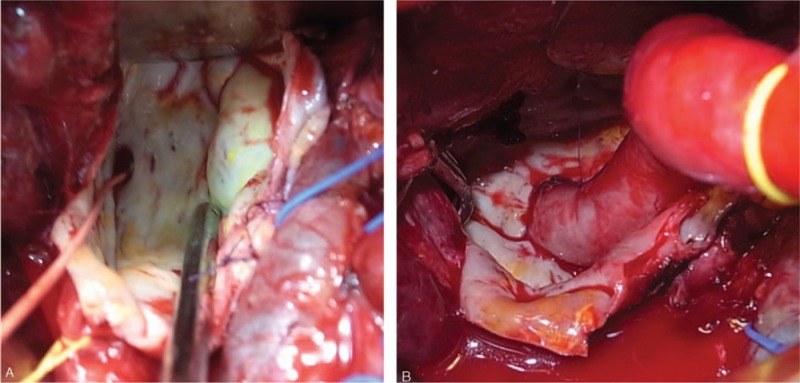
(A) The larger right HAA was opened and then back bleeding was controlled using a 2F Fogarty catheter. (B) Reconstruction was achieved by anastomosing the distal orifice and the right hepatic artery using the inclusion technique. HAA = hepatic artery aneurysm.

During the operation, an initial angiogram was obtained (Fig. 2A). Following laparotomy, the larger right HAA was found in the liver bed, compressing the right robe. By exposing the hepatic artery system, the other 2 HAAs were also identified. Then, the A6 off the smaller right HAA was ligated. After intravenous heparin was given, the right hepatic artery was clamped and the larger right HAA was then opened without distal control. Because the larger right HAA was deeply embedded within liver parenchyma, the outflow was identified from inside the aneurysm following the aneurysmotomy and a 2F Fogarty balloon catheter was placed into the outflow in order to control the back bleeding (Fig. 3A). The right hepatic artery just proximal to the smaller right HAA was transected and anastomosed directly to the distal orifice inside the larger right HAA in an end-to-end fashion with running 5-0 polypropylene, using the inclusion technique (Fig. 3B). Resection of the common HAA was done, after the GDA was ligated. Then, an end-to-end anastomosis of the proximal and distal edges of the common hepatic artery was performed. Finally, a completion angiogram showed the absence of the 3 aneurysms and that the hepatic blood flow was maintained without anastomotic stenosis or hepatic artery kinking (Fig. 2B).

The operative time was 398 minutes. The blood loss was 256 mL and the cell saver blood returned was 1100 mL without the need of blood transfusion. The postoperative course was uneventful. The maximum level of transaminase was that on postoperative day 1 (aspartate transaminase, 111 U/L and alanine transaminase, 65 U/L), and transaminase levels recovered to the baseline on postoperative day 7. She was discharged from the hospital on postoperative day 13.

A follow-up CT scan 2 months after surgery confirmed a patent distal anastomosis (arrow) without any findings of hepatic atrophy or necrosis. The intrahepatic bile duct dilatation in the right lobe was also resolved, along with an improvement of the hepatic enzymes (Fig. 1B). As of her last follow-up visit, 1 year after surgery, the patient was doing well without any symptoms.

## 3 Discussion

Liver involvement in HHT is defined by a spectrum of VMs. Given the dual blood supply to the liver, 3 different patterns of intrahepatic shunting can be observed: arteriovenous, arterioportal, and/or portovenous malformations. Most hepatic VMs in HHT patients are asymptomatic and rarely become symptomatic. Depending on shunt size and patterns, intrahepatic shunts can lead to the 3 most common clinical presentations: high-output cardiac failure, portal hypertension, and biliary disease.^[2]^ No treatment is recommended for patients with asymptomatic liver involvement in HHT, whereas in patients with symptomatic liver involvement, intensive medical therapy is indicated for these life-threatening complications. Liver transplantation should be considered only in patients who fail to respond to such therapy.^[7]^

There are other treatment options that aim at reducing shunts, such as ligation or embolization. However, these procedures should be limited to high-risk candidates for liver transplantation,^[7]^ because the mortality rates related to these procedures are extremely high, ranging up to 20%.^[8]^ Several authors^[2,6]^ have reported excessive hepatic necrosis after hepatic artery ligation, probably because the portal vein flow was inadequate due to the presence of portovenous shunts. Notably, the most common reason for liver transplantation has been postprocedural complication or recurrence resulting from shunt-reducing procedures.^[8]^ In terms of hepatectomy in patients with hepatic HHT, no consensus exists because of the lack of sufficient clinical data, although 3 cases have been published in the literature.^[9,10]^

When treating HAAs, there are a variety of options including open surgery (ligation, reconstruction, and hepatectomy) and endovascular treatment (embolization and stent-grafting). It is clear that hepatic VMs affect the patients in various ways postprocedurally. Even if VMs are asymptomatic before procedure, symptoms related to VMs might emerge due to the effect of the procedure. With this in mind, we believe that arterial reconstruction can play an important role in the treatment of HHT-associated HAAs because of minimal hemodynamic changes in the liver.

In our case, some parts of this intrahepatic aneurysm were exposed to the peritoneal cavity. Because dissecting between the aneurysmal wall and the liver parenchyma may lead to bleeding and biliary injury, we opened the aneurysm without obtaining distal control and back bleeding was controlled from inside the aneurysm. Stent grafting was not an option due to the extreme tortuosity of the hepatic arteries and due to the absence of distal landing zone in the right hepatic artery. On the other hand, we did not need bypass grafts because the hepatic arteries were elongated and mobilization of the native arteries provided sufficient length. Otherwise, we would use saphenous vein graft.

If reconstruction had been impossible, we planned to perform a right hepatectomy. However, the remaining left lobe might have become hyperperfusive postoperatively, possibly leading to increased blood flow in the intrahepatic shunts and subsequent adverse events. In this respect, we believe that benefits from obviating hepatectomy were significant.

It remains unclear whether the standard 2 cm-threshold for surgical treatment can be applied in patients with HTT-associated HAAs, as it is for ordinary visceral aneurysms. However, it is clear that one should weigh the risk of aneurysmal rupture against the other conditions of the disease.

## 4 Conclusions

We believe that arterial reconstruction can play an important role in the treatment of HHT-associated HAAs. We also believe that arterial reconstruction might reduce the risk of postprocedural complications with minimal hemodynamic changes, thus obviating the need for hepatectomy or liver transplantation.

## References

[1] Guttmacher AE Marchuk DA White RI Jr Hereditary hemorrhagic telangiectasia. *N Engl J Med* 1995; 333:918–924. 7666879 10.1056/NEJM199510053331407

[2] Garcia-Tsao G Korzenik JR Young L Liver disease in patients with hereditary hemorrhagic telangiectasia. *N Engl J Med* 2000; 343:931–936. 11006369 10.1056/NEJM200009283431305

[3] Miyabe K Akita S Kitajima Y Rupture of hepatic aneurysm complicating hereditary hemorrhagic telangiectasia (Osler–Weber–Rendu disease) for which hepatic arterial coil embolization was effective. *J Gastroenterol Hepatol* 2007; 22:2352–2357. 18031399 10.1111/j.1440-1746.2006.03456.x

[4] Chou YH Tiu CM Hsu CC Hereditary haemorrhagic telangiectasia: hepatic lesions demonstrated with colour Doppler and power Doppler sonography. *Eur J Radiol* 2000; 34:52–56. 10802208 10.1016/s0720-048x(99)00090-x

[5] Condon JR Tanner NC Cowper DM Hepatic artery aneurysm, hereditary haemorrhagic telangiectasia, and peptic ulceration. *Gut* 1967; 8:377–379. 6039727 10.1136/gut.8.4.377 PMC1552538

[6] Graham WP III Eiseman B Pryor R Hepatic artery aneurysm with portal vein fistula in a patient with familial hereditary telangiectasia. *Ann Surg* 1964; 159:362–367. 14129379 PMC1408598

[7] Buscarini E Plauchu H Gracia Tsao G Liver involvement in hereditary hemorrhagic telangiectasia: consensus recommendations. *Liver Int* 2006; 26:1040–1046. 17032403 10.1111/j.1478-3231.2006.01340.x

[8] Lerut J Orlando G Adam R Liver transplantation for hereditary hemorrhagic telangiectasia. Report of the European Liver Transplant Registry. *Ann Surg* 2006; 244:854–864. 17122610 10.1097/01.sla.0000247258.35406.a4 PMC1856634

[9] D’Angelo M Baiocchi L Lerut J Non-transplant surgical approach to liver-based gereditary haemorrhagic telangiectasia: a first report. *Liver Int* 2008; 28:574–577. 17900244 10.1111/j.1478-3231.2007.01588.x

[10] Gaujoux S Bucau M Ronot M Liver resection in patients with hepatic hereditary hemorrhagic telangiectasia. *Dig Surg* 2013; 30:410–414. 24217369 10.1159/000351446

